# Efficacy and survival of anti-PD-1 antibody in combination with trastuzumab and chemotherapy versus trastuzumab and chemotherapy as first-line treatment of HER2-positive metastasis gastric adenocarcinoma: a retrospective study

**DOI:** 10.3389/fonc.2023.1166040

**Published:** 2023-05-18

**Authors:** Ting Deng, Danyang Li, Yuchong Yang, Feixue Wang, Ming Bai, Rui Liu, Hongli Li, Yi Ba

**Affiliations:** Department of GI Medical Oncology, Tianjin Medical University Cancer Institute and Hospital, National Clinical Research Center for Cancer, Tianjin’s Clinical Research Center for Cancer, Key Laboratory of Cancer Prevention and Therapy, Tianjin, China

**Keywords:** gastric/gastroesophageal adenocarcinoma, HER2-positive, anti-PD-1 antibody, trastuzumab, chemotherapy

## Abstract

**Background:**

The KEYNOTE-811 study exhibited promising preliminary results for HER2-positive metastasis gastric adenocarcinoma; however, long-term survival benefit remains to be determined.

**Methods:**

In this single-center, controlled, retrospective study, patients with histologically confirmed HER2-positive unresectable or metastatic gastric/gastroesophageal adenocarcinoma received either anti-PD-1 antibody combined with trastuzumab and chemotherapy (cohort A) or trastuzumab and chemotherapy treatment (cohort B). The primary end points were progression-free survival (PFS) and overall survival (OS). The secondary end points were objective response rate (ORR), disease control rate (DCR), and duration of response (DoR).

**Results:**

A total of 56 patients were eligible to join the study, with 30 patients in cohort A and 26 patients in cohort B. The median PFS (mPFS) was 16.2 months (95% CI, 15.093–17.307) in cohort A versus 14.5 months (95% CI, 9.491–19.509) in cohort B (*p* = 0.58). The median OS in cohort A was 28.1 months (95% CI, 17.625–38.575) versus 31.6 months (95% CI, 13.757–49.443) in cohort B (*p* = 0.534). ORRs were 66.7% and 50% in the two groups, respectively. DCRs were 90% and 84.6% in the two groups. Median DoR was not reached in cohort A and it was 16.3 (95% CI, 8.453–24.207) months in cohort B (*p* = 0.141). The most common irAEs were grade 1 hypothyroidism (33.3%) in cohort A. No treatment-related deaths occurred in this study.

**Conclusions:**

This retrospective cohort study provided a preliminary picture on the long-term follow-up of combining anti-PD-1 antibody with trastuzumab and chemotherapy in HER2-positive GC, and a trend with longer DoR and ORR was identified. Further studies with larger sample sizes and more in-depth molecular investigation are needed.

## Introduction

Gastric cancer is the fifth most common cancer and the fourth leading cause of cancer-related death worldwide in 2020 (1). In China, advanced diseases account for nearly 80%, and the prognosis remains poor with a 5-year survival rate of less than 30% (2, 3). Over the last decades, targeted therapy and immunotherapy emerge as revolutionary breakthroughs in cancer treatment. The success of the ToGA trial shed light on HER2-targeted therapy in gastric cancer. The transmembrane receptor erbB-2, also known as human epidermal growth receptor 2 (HER2), is a member of the epidermal growth factor family of receptor tyrosine kinases (4). Overexpression or amplification of the HER2 gene or protein has been implicated in the development of gastric adenocarcinoma (5). In the ToGA trial, the proportion of HER2-positive patients [defined as immunohistochemistry (IHC) 3+ regardless of fluorescence *in situ* hybridization (FISH) status, or IHC 2+ and FISH positive] was approximately 16.6% (6), while in Chinese patients, the positivity rate is lower, approximately 12.0% (7).

For HER2-positive advanced gastric cancer patients, the addition of trastuzumab to traditional chemotherapy in a first-line setting can obtain a median overall survival (mOS) of 13.8 months, a big step in gastric cancer treatment (6). Given the positive result of the ToGA trial, subsequent related studies (HERBIS-1, WJOG7212G, CGOG1001, and KSCC trial) on trastuzumab were conducted and presented similar results (8). Moreover, other forms of single or combinational pattern of trastuzumab were also tried. Single targeted therapy has shown limited clinical efficacy. The JACOB trial adding pertuzumab, another anti-HER2 antibody, to trastuzumab and chemotherapy has shown improved progression-free survival (PFS); however, no significant difference was observed in the primary end point OS (9).

Recently, immune checkpoint inhibitors were found to be efficacious in various tumor types, including gastric cancers (10). Previous studies have confirmed that trastuzumab inHER2-positive gastric cancer patients can modulate the tumor microenvironment (TME), *via* upregulating PD-1 and PD-L1 expression, expansion of tumor-specific CD4 and CD8 T cells, and enhancing the antitumor effects of immune checkpoint inhibitors (11, 12). The randomized, double-blind, placebo-controlled phase III KEYNOTE-811 study investigated the safety and efficiency of the combinational form of anti-PD-1 antibody with trastuzumab and chemotherapy, and the objective response rate (ORR) and disease control rate (DCR) reached 74.4% and 96.2%, respectively, in the pembrolizumab group. This study opened the possibility that combining pembrolizumab with trastuzumab and chemotherapy can further improve the survival of HER2-positive gastric cancer patients (13), whereas the long-term survival was unclear. Therefore, we conducted a retrospective study to compare the efficacy and survival of trastuzumab combined with chemotherapy versus anti-PD-1 antibody combined with trastuzumab and chemotherapy in patients with HER2-positive gastric adenocarcinoma.

## Methods

### Study design and participants

This is a single-center, controlled, retrospective study carried out at Tianjin Medical University Cancer Institute and Hospital. Patients diagnosed with HER2-positive unresectable or metastasis gastric/gastroesophageal (GC/GEJ) adenocarcinoma and treated between January 2015 and September 2021 were enrolled.HER2-positive was defined as IHC 3+ or FISH positive regardless of IHC status. Inclusion criteria were as follows: men or women older than 18 years; Eastern Cooperative Oncology Group (ECOG) performance status of 0–2; with measurable diseases; receiving at least one cycle of different treatment; and having an expected survival time over 12 weeks. Previous chemotherapy and any antitumor therapy for metastatic disease were major exclusion criteria except receiving adjuvant or neoadjuvant treatment over 6 months. Patients with autoimmune diseases or receiving immunotherapy were also excluded.

### Treatment and outcome

Patients treated with anti-PD-1 antibody combined with trastuzumab and chemotherapy were assigned to cohort A, whereas those receiving trastuzumab and chemotherapy were assigned to cohort B. Trastuzumab was given by intravenous infusion at a dose of 8 mg/kg on day 1 of the first cycle, followed by 6 mg/kg every 3 weeks, or at a dose of 6 mg/kg on day 1 of the first cycle, followed by 4 mg/kg every 2 weeks. The dosages of anti-PD-1 antibody varies in different drugs (nivolumab and toripalimab 240 mg intravenously; pembrolizumab, sintilimab, tislelizumab, and camrelizumab 200 mg intravenously every 2 or 3 weeks). Chemotherapy regimen was selected by investigators according to National Comprehensive Cancer Network (NCCN) guidelines. Maintenance treatment was the triple regimen trastuzumab, anti-PD-1 antibody, and oral fluorouracils (capecitabine or S-1) repeated every 3 weeks in cohort A. In cohort B, patients received the double regimen trastuzumab and oral fluorouracils in the maintenance phase every 3 weeks. Treatment continued up to 2 years of disease progression, and intolerable toxicity occurred within 2 years in the two cohorts.

Tumor assessment was performed by imaging according to Response Evaluation Criteria in Solid Tumors (RECIST), version 1.1. Adverse events (AEs) were evaluated by the National Cancer Institute Common Terminology Criteria for Adverse Events (CTCAE), version 4.0. The primary end points were PFS and OS. PFS was defined as the time from first cycle treatment to first disease progression or death and OS was defined as time from first cycle treatment to death. The secondary end points were ORR (complete or partial response), DCR (complete, partial, and stable disease), and duration of response (DoR). DoR was defined as the date of first objective response to the date of first progressive disease or death. In addition, we performed stratified analysis on patient characteristics (including sex, ECOG, tumor location, and HER2 status) by treatment arm. Moreover, we collected the numbers of neutrophils and lymphocytes at baseline and calculated the neutrophil-to-lymphocyte ratio (NLR) of enrolled patients. NLR was defined as neutrophil count (number/L)/lymphocyte count (number/L).

### Statistical analysis

Qualitative variables were described using frequency and percentage, and continuous variables were reported using median and range. PFS, OS, and DoR were assessed using the Kaplan–Meier method, described with median and 95% confidence interval (CI). Survival analyses between groups were conducted using the log-rank test. Statistical analysis was performed with SPSS (version 24.0). All tests were two-sided, and *p* < 0.05 was considered statistically significant.

## Results

### Patient characteristics

**Figure 1:**
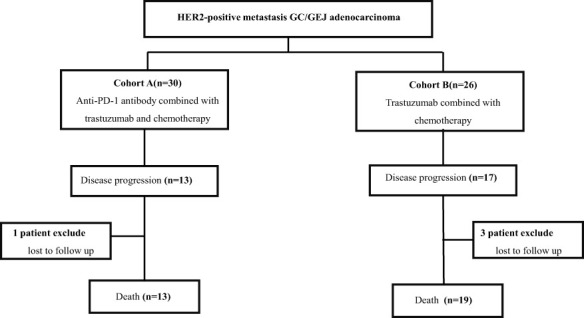
Trial profile. Patients with HER2-positive unresectable or metastasis gastric/gastroesophageal (GC/GEJ) adenocarcinoma separated in two groups. Cohort A received anti-PD-1 antibody combined with chemotherapy and trastuzumab; cohort B received chemotherapy combined with trastuzumab.

From October 2015 to August 2021, a total of 56 HER2-positive metastasis GC/GEJ patients were eligible to join this study. Thirty patients received anti-PD-1 antibody combined with chemotherapy and trastuzumab, and 26 patients received trastuzumab plus chemotherapy. The trial profile is shown in **Figure 1**. The median age was 62 years (23–77 years) in cohort A, including 22 men (73.3%) and 8 women (26.7%). In cohort B, the median age was 55.5 years (30–76 years), with 18 men (69.2%) and 8 women (30.8%). In cohort A, 15 patients (50%) had IHC 3+, and 25 patients received oxaliplatin-based chemotherapy regimen, including SOX (oxaliplatin 130 mg/m² intravenous on day 1 plus oral S-1 twice a day for 2 weeks followed by 1 week’s rest; S-1 was dependent on body surface area: body surface area >1.5 m², 60 mg twice a day, body surface area 1.25–1.5 m², 50 mg twice a day, body surface area <1.25 m², 50 mg twice a day), CapOx (oxaliplatin 130 mg/m² intravenous on day 1 plus oral 1,000 mg/m² capecitabine twice a day for 2 weeks, followed by 1 week of rest), and FOLFOX (oxaliplatin 85 mg/m^2^ intravenous and leucovorin 400 mg/m^2^ intravenous, followed by 5-fluorouracil 400 mg/m^2^ intravenous, and then 2.4 mg/m^2^ as 46 h of continuous intravenous infusion on day 1 of each cycle). In cohort B, 18 patients (69.2%) had IHC 3+, and 20 patients (76.9%) received oxaliplatin-based chemotherapy. The baseline characteristics of patients are summarized in **Table 1**.

**Table 1:** The baseline patient characteristics.

Variables	Cohort A (*n* = 30)	Cohort B (*n* = 26)
**Median age, years (range)**	23–77 (62)	30–76 (55.5)
Sex, *n* (%)		
Male	22 (73.3%)	18 (69.2%)
Female	8 (26.7%)	8 (30.8%)
ECOG performance score, *n* (%)		
0	6 (20%)	5 (19.2%)
1	24 (80%)	19 (73.1%)
2	0 (0%)	2 (7.7%)
Histologic type, *n* (%)		
Adenocarcinoma	28 (93.3%)	25 (96.2%)
Signet-ring cell carcinoma	2 (6.7%)	1 (3.8%)
Primary tumor location, *n* (%)		
Gastro-esophageal junction	5 (16.7%)	10 (38.5%)
Gastric	24 (80%)	15 (57.7%)
Unknown	1 (3.3%)	1 (3.8%)
Chemotherapy, *n* (%)		
Oxaliplatin based	25 (83.3%)	20 (76.9%)
Paclitaxel based	3 (10%)	2 (7.7%)
Others	2 (6.7%)	4 (15.4%)
Median cycles of treatment		
First-line treatment	7 (1–29)	10 (1–32)
Anti-PD-1 antibody	5 (1–29)	
Trastuzumab	7 (1–29)	8 (1–30)
Chemotherapy	6 (1–29)	10 (1–32)
**Previous gastrectomy, *n* (%)**	6 (20%)	8 (30.8%)
HER2 status, *n* (%)		
FISH positive/IHC 1+	1 (3.3%)	0 (0%)
FISH positive/IHC 2+	12 (40%)	6 (23.1%)
FISH positive/IHC 0/Unknown	2 (6.7%)	2 (7.7%)
IHC 3+	15 (50%)	18 (69.2%)
EBV status, *n* (%)		
Positive	0 (0%)	0 (0%)
Negative	18 (60%)	9 (34.7%)
Unknown	12 (40%)	17 (65.4%)
MMR status, *n* (%)		
dMMR	0 (0%)	0 (0%)
pMMR	23 (76.7%)	14 (53.8%)
Unknown	7 (23.3%)	12 (46.2%)

### Efficacy and survival

**Figure 2:**
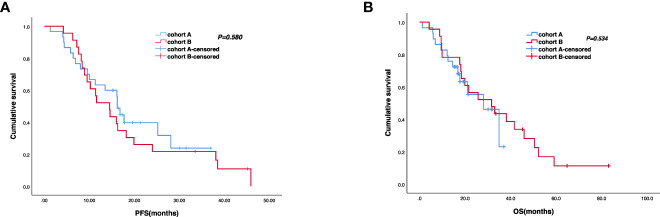
Kaplan–Meier analyses of progression-free survival **(A)** (cohort A, *n* = 30; cohort B, *n* = 23) and overall survival **(B)** (cohort A, *n* = 29; cohort B, *n* = 23) of the two groups.

**Figure 3:**
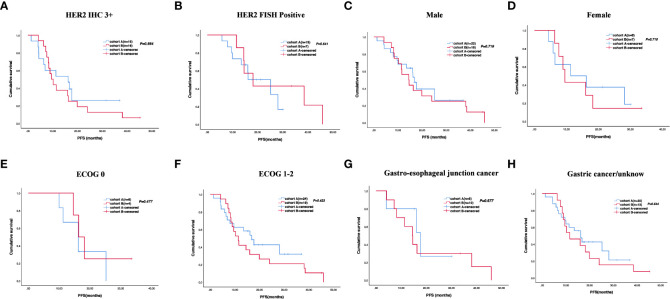
Stratified analysis of progression-free survival in different subgroups, including HER2 status **(A, B)**, sex **(C, D)**, ECOG **(E, F)**, and tumor location **(G, H)**.

**Figure 4:**
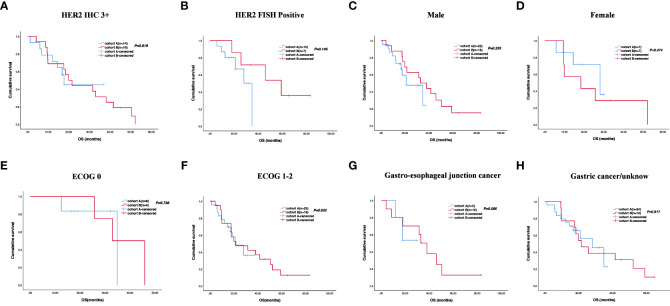
Stratified analysis of overall survival in different subgroups, including HER2 status **(A, B)**, sex **(C, D)**, ECOG **(E, F)**, and tumor location **(G, H)**.

**Figure 5:**
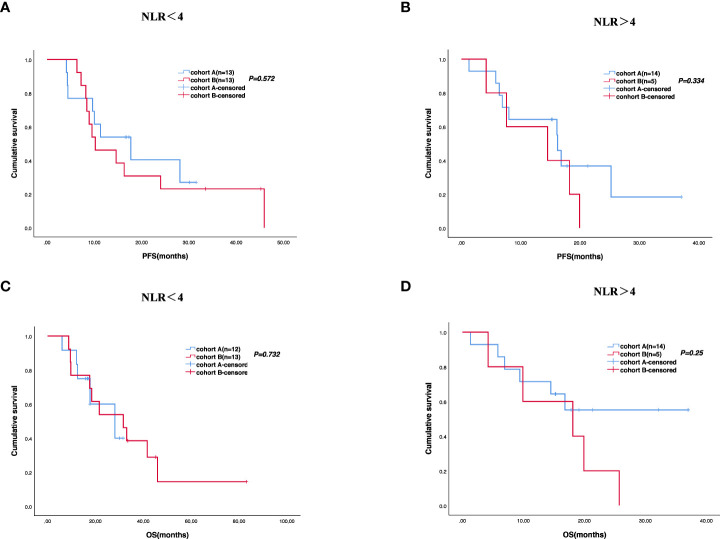
Survival difference of neutrophil-to-lymphocyte ratio (NLR) of the two groups. Progression-free survival **(A, B)** and overall survival **(C, D)**.

The cutoff follow-up date for this study was 1 November 2022. The median follow-up time was 17.05 months (IQR: 1.3–37) and 23.65 months (IQR: 3.07–83) in cohorts A and B respectively. A total of 13 (43.3%) patients in cohort A and 17 (65.4%) in cohort B experienced disease progression. One patient in cohort A and three patients in cohort B were lost to follow-up. The median PFS (mPFS) was 16.2 months (95% CI, 15.093–17.307) in cohort A versus 14.5 months (95% CI, 9.491–19.509) in cohort B (*p* = 0.58) (**Figure 2A**). The 1-year PFS rate of cohorts A and B was 63.3% and 52.2%, respectively. The 2-year PFS rate of the two groups was 43.3% and 21.3%, respectively. At the cutoff date, 32 deaths occurred, including 13 deaths in cohort A and 19 in cohort B. The median overall survival time in cohort A was 28.1 months (95% CI, 17.625–38.575) in comparison with 31.6 months (95% CI, 13.757–49.443) in cohort B (*p* = 0.534) (**Figure 2B**). The 1-year OS rate was 82.8% and 78.3% in cohorts A and B, respectively, and the 2-year OS rate was 62.1% and 56.5% in the two groups. The stratified analysis of patient characteristics (including sex, ECOG, tumor location, and HER2 status) and NLR showed no statistically significant difference in survival from different subgroups (**Figures 3**–**5**). For patients with disease progression, 4 (30.1%) in cohort A and 10 (58.8%) in cohort B received more than second-line therapy. The later line treatment details of two groups are shown in **Table S1**.

**Figure 6:**
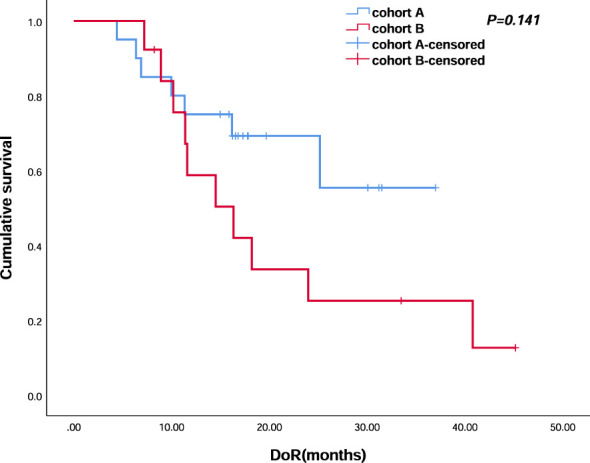
Kaplan–Meier analyses of duration of response (cohort A, *n* = 30; cohort B, *n* = 23) of the two groups.

All 56 patients received at least one cycle of the first-line treatment regimen and completed efficacy evaluation. No CR was observed in cohort A, and one patient (3.8%) experienced CR in cohort B. Twenty patients (66.7%) evaluated PR in cohort A and 12 patients (46.2%) evaluated PR in cohort B. Seven patients (23.3%) evaluated SD in cohort A and nine (34.6%) in cohort B. ORR was 66.7% and 50%, respectively. DCR was 90% and 84.6% in the two groups, respectively. The objective response of enrolled patients is shown in **Table 2**. Median DoR was not reached (NR) in cohort A and was 16.3 (95% CI, 8.453–24.207) months in cohort B (*p* = 0.141) (**Figure 6**).

**Table 2:** Summary of confirmed objective response in enrolled patients.

Variable	Cohort A (*n* = 30)	Cohort B (*n* = 26)
**Objective response rate (ORR) (%)**	66.70%	50.00%
**Disease control rate (DCR) (%)**	90%	84.60%
Complete response (CR)	0 (0%)	1 (3.8%)
Partial response (PR)	20 (66.7%)	12 (46.2%)
Stable disease (SD)	7 (23.3%)	9 (34.6%)
Progressive disease (PD)	1 (3.3%)	2 (7.7%)
Not assessed	2 (6.7%)	2 (7.7%)

### Safety

All patients in this study experienced at least one treatment-related adverse event (TRAE); 40% in cohort A and 46.1% in cohort B had AEs of grades 3–4. There was no significant difference between the two cohorts on the rate of grade 3–4 AEs, except for bilirubin increased and skin reaction. In cohort A, 10 patients experienced immune-related adverse effects (irAEs), among which the most common one was hypothyroidism (33.3%). All the hypothyroidism cases were grade 1. Among the 10 patients developing hypothyroidism, 1 patient had grade 2 pneumonia, 1 patient had grade 2 myocarditis, and 1 patient had grade 3 skin reaction at the same time. After treatment, all the three patients recovered completely. No treatment-related treatment discontinuation or deaths occurred in the two groups. No statistically significant differences in the incidence of AEs were noted between the two groups (**Table 3**).

**Table 3:** The major adverse events (AEs).

	Grades 1–2 (%)		Grade 3 (%)		Grade 4 (%)	
	Cohort A (*n* = 30)	Cohort B (*n* = 26)	Cohort A (*n* = 30)	Cohort B (*n* = 26)	Cohort A (*n* = 30)	Cohort B (*n* = 26)
Hematologic system						
Anemia	12 (40%)	9 (34.7%)	4 (13.3%)	5 (19.2%)		
Neutropenia	12 (40%)	9 (34.7%)	4 (13.3%)	3 (11.5%)		2 (7.7%)
Leukopenia	17 (56.7%)	10 (38.5%)		1 (3.8%)		
Thrombocytopenia	10 (33.3%)	10 (38.5%)	1 (3.3%)			1 (3.8%)
Alimentary system						
Nausea	1 (3.3%)	3 (11.5%)				
Vomit		2 (7.7%)				
Loss of appetite	4 (13.3%)	4 (15.4%)				
Ventosity	1 (3.3%)	3 (11.5%)				
Bilirubin increased	3 (10%)		1 (3.3%)			
ALT increased	8 (26.7%)	10 (38.5%)				
AST increased	10 (33.3%)	8 (30.8%)				
Hypoalbuminemia	3 (10%)	7 (26.9%)				
Endocrine and metabolic system						
Hypokalemia	3 (10%)	1 (3.8%)				
Hyponatremia	3 (10%)	4 (15.4%)				
Others						
Peripheral neurotoxicity		1 (3.8%)	1 (3.3%)			
Fever	3 (10%)	4 (15.4%)				
Bleached	2 (6.7%)	2 (7.7%)				
Shingles	1 (3.3%)					
Pain		2 (7.7%)				
Immunotherapy related						
Hypothyroidism	10 (33.3%)					
Pneumonia	1 (3.3%)					
Myocarditis	1 (3.3%)					
Skin reaction			1 (3.3%)			

## Discussion

In this retrospective study, we provided the initial picture of the comparison on different treatment patterns as trastuzumab plus chemotherapy with or without anti-PD-1-based immunotherapy in HER2 positive GC/GEJ adenocarcinoma from one single center. Since the ToGA trial brought gastric cancer treatment into the new era, there was little breakthrough in the last decade. In a retrospective analysis on the ATTRACTION-2 trial, researchers compared the survival parameters of HER2 positive and -negative subgroups, observing a significantly improved OS (8.3 vs. 4.8 months, *p* < 0.05) of HER2 positive patients using nivolumab (14). This result suggested that HER2 positive gastric cancer patients who have previously used trastuzumab may benefit from anti-PD-1 antibody. AIO INTEGA trial evaluated the efficacy of ipilimumab vs. FOLFOX in combination with nivolumab and trastuzumab in previously untreated ERBB2-positive esophagogastric adenocarcinoma (EGA), and the FOLFOX group showed better survival data with an ORR of 63% and a 1-year OS rate of 70%, indicating that at the current stage, chemotherapy removal is not an advisable choice (15). All these previous data prompted the investigation on combining immunotherapy with a traditional first-line treatment setting in HER2-positive GC patients.

In our study, the treatment cohort with anti-PD-1 therapy achieved a DCR of 90%, which was comparable to the phase III KEYNOTE-811 study (13). The ORR was 66.7%, lower than that reported from KEYNOTE-811, which may be related to the small sample size of our study. Although no obvious differences were observed in PFS and OS between the two cohorts in our trial, the 2-year PFS rate was significantly higher in cohort A than in cohort B (40% and 21.3%). Moreover, a longer DoR was identified (NR vs. 16.3 months, *p* = 0.141), the improvement of which is not so outstanding in the KEYNOTE 811 study (10.6 months vs. 9.5 months). We compared the incidence of AEs between the two groups. The addition of immunotherapy did not increase the AEs as the incidence of grade 3–4 AEs was 40% and 46.1% in cohorts A and B, respectively. Furthermore, no grade 3 or 4 immune-related AEs occurred in cohort A. All these results suggested that the combination with immunotherapy is clinically safe and could be a treatment option for HER2-positive advanced GC or GEJ adenocarcinoma patients.

Blockade of immune checkpoints exerts antitumor effect *via* amplifying the antitumor immune responses (16). Results of CheckMate-649 suggest that the combination of immune checkpoint inhibitor and chemotherapy significantly improves OS of advanced GC (17). In addition, nivolumab combined with oxaliplatin-based chemotherapy demonstrated significantly improved PFS in the ATTRACTION-4 study (18). Combination therapy is currently a major direction for cancer treatment. Basic research also verified that combining with anti-PD-1 antibody could obviously boost the therapeutic activity of the anti-ErbB-2 monoclonal antibody (mAb) in BALB/c-ErbB-2 transgenic mice (19). Moreover, trastuzumab could enhance the efficacy of immune checkpoint inhibitors through increasing HER2 internalization and cross-presentation by dendritic cells (20), upregulating PD-1 and PD-L1 expression, inducing the expansion of tumor-specific CD4 and CD8 T cells and MHC class II expression (11, 12, 21). This provides a theoretical basis for combination therapy. Consistent conclusions were also observed in HER2 positive breast cancer, as combined immunotherapy could demonstrate clinical efficiency. Several studies have demonstrated that immunotherapy combined with anti-HER2 treatment and chemotherapy in a neoadjuvant setting can achieve an acceptable pCR rate (22, 23), while single-agent immunotherapy showed limited efficacy compared with endocrine treatment and targeted therapy, which are now the mainstay treatment for this group of breast cancer patients (24).

Aside from KEYNOTE-811, several other clinical trials targeting both HER2 and immunotherapy were also conducted in GC. A phase Ib/II trial evaluated the antitumor activity of margetuximab (the Fc-optimized anti-HER2 monoclonal antibody) plus pembrolizumab in 95 previously treated HER-2-positive gastric or gastroesophageal junction (GEJ) adenocarcinoma patients (25). ORR was 18.48% and DCR was 53%. The mPFS and mOS were 2.73 months and 12.48 months, respectively. Further subgroup analysis demonstrated that objective response was most pronounced in patients with HER2 IHC 3+ and PD-L1-positive tumors (26). The phase II/III MAHOGANY trial including three arms, margetuximab plus retifanlimab (anti-PD-1 mAb) with or without chemotherapy and margetuximab plus tebotelimab (anti-PD-1 and anti-LAG-3 bispecific mAb) with chemotherapy in first-line unresectable metastatic/locally advanced GEJ adenocarcinoma is ongoing (NCT04082364) (27). Zanidatamab (ZW25, a new bispecific antibody simultaneously binding two non-overlapping epitopes of HER2) (28) has already demonstrated clinical benefits with an ORR of 75% and a median DoR of 16.4 months when combined with chemotherapy (CAPOX, CF, or mFOLFOX6) in the first-line treatment (29). Thus, another ongoing study further explored the antitumor activity of ZW25 in combination with chemotherapy and anti-PD-1 antibody (NCT04276493). Similarly, another phase Ib study was performed to evaluate the clinical efficiency of KN026, another bispecific antibody targeting HER2 (30) and KN046 (an anti-PD-L1 and anti-CTLA4 bispecific antibody) for metastatic GC and GEJ adenocarcinoma. Results identified an ORR of 86% in the first-line cohort (NCT04040699) (31). These studies all suggested that combining HER2-targeted therapy with immunotherapy could improve the antitumor response of advanced GC/GEJ adenocarcinoma patients.

In this retrospective cohort study, compared with the current standard therapeutic regimen, the addition of anti-PD-1 immunotherapy did not significantly improve the PFS or OS, for which the small sample size and short follow-up time may be the possible reasons. However, a trend of higher ORR and longer DoR was identified; moreover, as shown in **Figure 6**, we observed an intersection in the early stage and obvious separation in late stage. Thus, the long-term follow-up and precise population selection are warranted to further assess the clinical transformation.

Recently, a multi-center phase II trial evaluated the safety and efficacy of quadruplet regimen (pembrolizumab, trastuzumab, and doublet chemotherapy) as first-line therapy for unresectable or metastatic HER2-positive advanced GC. ORR was 76.7% with CR and PR reported in 14% and 62.8% of patients, respectively. The mPFS was 8.6 months and mOS achieved 19.3 months. At the same time, they explored the molecular signatures of enrolled patients, and next-generation sequencing indicated that RTK/RAS pathway alterations were closely correlated with PFS (32). In our study, the OS of patients with HER2 FISH positive was different in two groups. However, these comparisons did not reach statistical significance due to the small sample size.

Limitations exist in this study, mainly originating from its retrospective design. Firstly, though no obvious difference in important parameters was observed between the two cohorts, some pathological and clinical parameters were not available for several cases. More detailed exploration of the molecular characteristics of HER2-positive patients is needed. Secondly, owing to the retrospective nature of the study, the immune drugs used in this study vary. Additionally, the number of patients in the two cohorts is not big enough. Further investigation with a larger cohort is needed.

With the development of immunotherapy in cancer, the combination of HER2-targeted drugs and immunotherapy is now a promising direction for the treatment of HER2-positive GC patients. The interim result from the phase III KEYNOTE-811 trial has already provided the preliminary evidence for this combination. In this study, we provided the initial long-term survival data from our center. We did not observe an obvious difference in PFS or OS, while an improved DoR and ORR was identified, indicating the potential of combining immunotherapy with the current standard regimen. Further randomized controlled investigation in a larger cohort is needed to determine the antitumor efficiency and final clinical transformation. In addition, a more in-depth molecular investigation is also critical to predict the populations that may benefit from the combined treatment.

## Conclusion

In this retrospective cohort study, we provided a preliminary picture on the long-term follow-up of combining anti-PD-1 with trastuzumab and chemotherapy in HER2-positive GC, although no statistically significant difference in PFS and OS was observed compared with the current standard. A trend with a longer DoR and ORR was identified, and further studies with larger sample sizes and a more in-depth molecular investigation are needed for clinical transformation.

## Data availability statement

The original contributions presented in the study are included in the article/**Supplementary Material**. Further inquiries can be directed to the corresponding authors.

## Ethics statement

The studies involving human participants were reviewed and approved by The Ethics Committee of Tianjin Medical University Cancer Institute and Hospital. The patients/participants provided their written informed consent to participate in this study.

## Author contributions

TD and YB were responsible for study concept and design. TD, DL, and FW were responsible for data analysis, data collection, and drafting and revision of the manuscript. TD and YY were responsible for imaging evaluation. TD, YY, MB, RL, and HL were responsible for the patient management. All authors contributed to the article and approved the submitted version.
