# Supplementary material for: Efficacy and survival of anti-PD-1 antibody in combination with trastuzumab and chemotherapy versus trastuzumab and chemotherapy as first-line treatment of HER2-positive metastasis gastric adenocarcinoma: a retrospective study
Source: Front Oncol. 2023 May 18;13:1166040. doi: 10.3389/fonc.2023.1166040 (PMC10234698; doi:10.3389/fonc.2023.1166040)
Supplement: Supplementary file 1 [file Table_1.docx]

**Supplement Table1.**

| **Table S1. Detailed information for later line treatment** | | |
| --- | --- | --- |
|  | **Cohort A** | **Cohort B** |
| **Disease progression** | 13 | 17 |
| **Afterwards therapy** |  |  |
| ≤second line | 9(69.2%) | 7(41.2%) |
| ＞second line | 4(30.1%) | 10(58.8%) |
| **Second-line treatment** |  |  |
| Irinotecan based | 3(23.1%) | 1(5.9%) |
| Paclitaxel based | 2(15.4%) | 5(29.4%) |
| Best supportive care | 5(38.5%) | 3(17.6%) |
| Combined with anti-PD-1 antibody | 4(30.8%) | 2(11.8%) |
| Combined with anti-HER2 treatment | 4(30.8%) | 8(47.1%) |
| Clinical trials | 0(0%) | 2(11.8%) |
| Others | 0(0%) | 1(5.9%) |
